# Physiological, Structural, and Functional Analysis of the Paralogous Cation–Proton Antiporters of NhaP Type from *Vibrio cholerae*

**DOI:** 10.3390/ijms20102572

**Published:** 2019-05-25

**Authors:** Muntahi Mourin, Alvan Wai, Joe O’Neil, Georg Hausner, Pavel Dibrov

**Affiliations:** 1 Department of Microbiology, University of Manitoba, Winnipeg, MB R3T 2N2, Canada; mourinm@myumanitoba.ca (M.M.); alvanwai@outlook.com (A.W.); Georg.Hausner@umanitoba.ca (G.H.); 2 Department of Chemistry, University of Manitoba, Winnipeg, MB R3T 2N2, Canada; Joe.ONeil@umanitoba.ca

**Keywords:** NhaP, cation–proton antiporters, *Vibrio cholerae*, acid tolerance response, molecular dynamics simulations

## Abstract

The transmembrane K^+^/H^+^ antiporters of NhaP type of *Vibrio cholerae* (Vc-NhaP1, 2, and 3) are critical for maintenance of K^+^ homeostasis in the cytoplasm. The entire functional NhaP group is indispensable for the survival of *V. cholerae* at low pHs suggesting their possible role in the acid tolerance response (ATR) of *V. cholerae*. Our findings suggest that the Vc-NhaP123 group, and especially its major component, Vc-NhaP2, might be a promising target for the development of novel antimicrobials by narrowly targeting *V. cholerae* and other NhaP-expressing pathogens. On the basis of Vc-NhaP2 in silico structure modeling, Molecular Dynamics Simulations, and extensive mutagenesis studies, we suggest that the ion-motive module of Vc-NhaP2 is comprised of two functional regions: (i) a putative cation-binding pocket that is formed by antiparallel unfolded regions of two transmembrane segments (TMSs V/XII) crossing each other in the middle of the membrane, known as the NhaA fold; and (ii) a cluster of amino acids determining the ion selectivity.

## 1. Introduction

*Vibrio cholerae* encodes for an elaborate set of membrane cation–proton antiporters that are responsible for the circulation of alkali cations Na^+^ and K^+^ [1,2]. These secondary ion pumps play a key role in ion and pH homeostasis. In particular, *V. cholerae* encodes three NhaP type antiporters, encoded by paralogous structural genes *Vc-nhaP1*, *2*, and *3*, mediating the exchange of K^+^ and Na^+^ for protons [3,4]. We found that all three Vc-NhaP-type antiporters exchange K^+^ for H^+^ in vivo and operate in concert to maintain the viability of *V. cholerae* cells in acidic, especially K^+^-rich environments [5]. Vc-NhaP2 seems to be a major component of this trio [5]. At the physiological level, the importance of the entire NhaP group for survival of *V. cholerae* at low pHs indicated its possible role at the critical step of the natural infectious process, when ingested *V. cholerae* passes the gastric acid barrier. Therefore, the Vc-NhaP123 group, and especially Vc-NhaP2, might be promising targets for the development of novel antimicrobials by narrowly targeting *V. cholerae* and other NhaP-expressing pathogens [5]. 

The NhaP type antiporters belong to the phylogenetically diverse cation/proton antiporter superfamily (CPA1) that includes the pharmacologically important NHEs, NHXs from plants, NhaP1 from archaea, and others [4,6,7,8,9,10,11,12,13]. The structural and functional analysis of prokaryotic antiporters has contributed to the overall understanding of human homologs important in health and disease [14]. For example, the human homolog of NhaP type antiporters, the NHE1 Na^+^/H^+^ exchangers, have been shown to be associated with different diseases, and a change in the activity of NHE1 plays role in heart failure [6]. It has been shown that chemical inhibition of NHE1 along with paclitaxel therapy resulted in a significant decrease in the invasive and migratory behaviour of breast cancer cells, indicating the potential of using NHE1 as a novel co-adjuvant target in combination with paclitaxel therapy to enhance the efficacy of breast cancer treatment [15]. Enhanced expression of NHE1 is also an indication of malignant gliomas [12]. Inhibitors against NHE1 significantly decreased the metastasis and enhanced the survival rate in mouse glioma models [12]. A structural model of clinically important human NHE1 was generated using the *E. coli* NhaA (Ec-NhaA) structure as a template to predict the binding site of NHE1 inhibitors [16]. In *Arabidopsis thaliana,* membrane trafficking, growth, cell expansion and internal pH homeostasis are maintained by NHXs K^+^(Na^+^)/H^+^ antiporters [13]. These NHXs antiporters share structural similarity especially in the core ion translocation domain with the bacterial Na^+^/H^+^ antiporters [13]. The Na^+^/H^+^ exchanger of *Schizosaccharomyces pombe* (Sp-NHE1) plays an important role in salt stress tolerance and mutation of some conserved acidic and polar amino acid residues located in either a transmembrane segment or an extracellular loop eliminated its ability to confer salt resistance [17]. The archaeal NhaP1 antiporters in *Pyrococcus abyssii* (Pa-NhaP1) and *Methanococcus jannaschii* (Mj-NhaP1) have been crystallized and widely studied [7,8,18,19]. The mammalian CPA1 antiporters are suggested to share sequence homology with the archaeal NhaP1 antiporters [20,21], and hence the crystal structures of Pa-NhaP1 and Mj-NhaP1 could be used as models for the transmembrane parts of NHE1 [19,21].

The extended and diverse NhaP family consists of transporters with different ion selectivities. It includes not only different bona fide Na^+^(Li^+^)/H^+^ antiporters such as the Mj-NhaP1 from the archeon *Methanococcus jannaschii* [7,8] and Pa-NhaP1 from *Pseudomonas aeruginosa* [8], but also cyanobacterial Syn-NhaP1, which possibly can use Ca^2+^ as another substrate [9], K^+^(Na^+^)/H^+^ antiporters Vc-NhaP1,2 [3,22,23], highly selective K^+^/H^+^ antiporter Vc-NhaP3 [5], Ca^2+^(Na^+^,Li^+^,K^+^)/H^+^ antiporter Yp-NhaP [10], and alkali-activated Aa-NhaP from *Alkalimonas amylolytica*, which can also exchange H^+^ for K^+^, Ca^2+^, and possibly NH_4_^+^, but not the smaller Li^+^ [11]. Together, therefore, they display a broad substrate specificity that includes the efflux of Li^+^, Na^+^ and K^+^. Of note, the NhaP family provided the first examples of truly Mitchellian K^+^/H^+^ antiporters in bacteria, which remained unidentified for a long time. Over the years, we have accumulated a considerable amount of biochemical information about Vc-NhaP2 [3,4,5,22,23,24]. Noticeably, the Vc-NhaP2 deletion mutant of *V. cholerae* is highly sensitive to external K^+^ at pH 6.0 although its resistance to Na^+^ is unaffected [3]. This observation suggests that in vivo Vc-NhaP2 acts as a K^+^/H^+^ antiporter, rather than a Na^+^/H^+^ antiporter [3]. Kinetic measurements of Vc-NhaP2 activity in the experimental model of everted sub-bacterial vesicles confirmed the ability Vc-NhaP2 to mediate direct K^+^/H^+^ exchange [3]. Results of the cation competition experiments carried out on Vc-NhaP2-expressing membrane vesicles have also strongly indicated that this antiporter is able to bind Li^+^ and countertransport it with Na^+^ or K^+^ but not H^+^ [3,4]. The chromosomal deletion of the *nhaP3* gene showed only minor growth defects at high potassium concentration at acidic pH [5]. Comparison of the biochemical properties of Vc-NhaP isoforms revealed that Vc-NhaP2 is the most active among all three paralogues with apparent K_m_ values for both K^+^ and Na^+^ of 1.6 and 1.04 mM respectively, whereas Vc-NhaP1 and Vc-NhaP3 demonstrated much weaker affinity to Na^+^ as well as K^+^ [3,5,23]. 

To explain these experimental data, we have suggested that protons, as well as alkali cations, all compete for different subsets of ligands within the common spacious ion-binding site of Vc-NhaP2 [4]. While H^+^ requires only one negative ligand for its coordination, the optimal coordination number for Li^+^ is 6 [25]. If the subset of ligands for Li^+^ happens to include a ligand used for protonation–deprotonation, the Li^+^ ion would always outcompete H^+^ and prevent direct Li^+^/H^+^ exchange. Our hypothesis implied that Li^+^ may directly or indirectly prevent H^+^ from binding to its ligand [4]. The molecular mechanism of this unusual ion selectivity of Vc-NhaP2 remains incompletely understood. An in-depth structural and functional analysis of Vc-NhaP2 could shed light on this intriguing problem. A detailed structural analysis would also illuminate the intra-molecular events comprising the catalytic cycle of NhaP-type antiporters.

## 2. Structural Model of Vc-NhaP2

**Figure 1:**
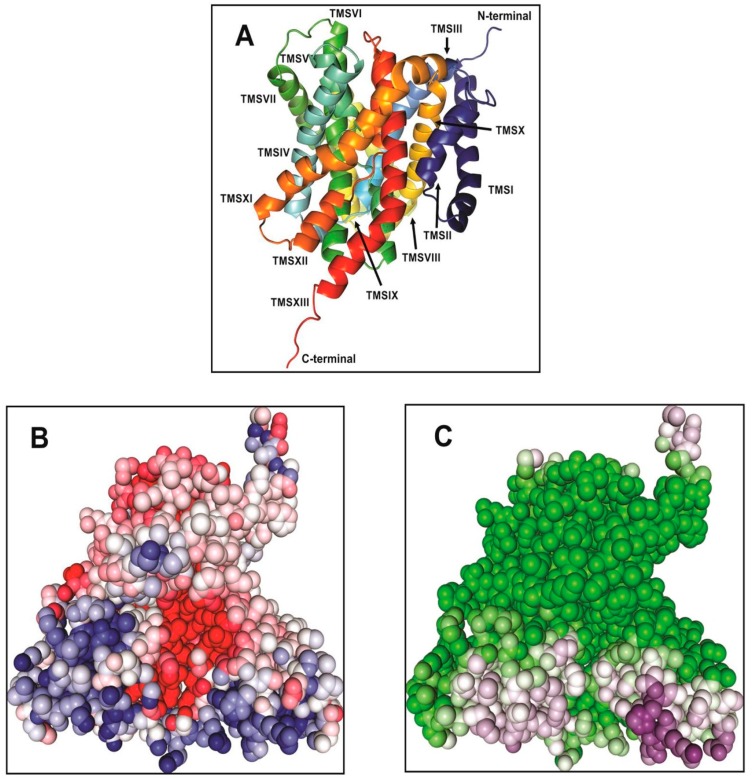
(**A**) The inward-open view of the structural model of the transmembrane domain of Vc-NhaP2. The structure was generated by Robetta and visualized by PyMOL 1.6 [34]. The model is shown in a rainbow of colors with TMSs numbered from I to XIII. (**B**) The electrostatic surface potential of Vc-NhaP2. A highly negatively charged (red colored) cavity is present in the middle of the Vc-NhaP2. The positively charged residues (blue colored) are lining the exterior of the antiporter. (**C**) The solvent accessible surface of Vc-NhaP2. The solvent accessible surface is indicated by the dark purple color. Solvent-inaccessible residues are green. The images for both electrostatic and solvent accessibility surface potential are generated by Protein-Sol (protein-sol.manchester.ac.uk) [35].

In our most recent study, we have combined homologue-based structure modelling with site-directed mutagenesis and antiport activity measurements to identify and characterize the structural elements responsible for the cation selectivity of Vc-NhaP2 [24]. The structural model for the transmembrane segments of Vc-NhaP2 that comprise the ion-motive functional module of the transporter was generated by *Phyre^2^* [26] and Robetta [27]. The *Pyrococcus abyssii* NhaP structure [18] was used as template to generate a structure by *Phyre^2^*. In the generated model, Vc-NhaP2 has an inward-open conformation (Figure 1). A negatively charged cavity is present in the middle of the membrane which is accessible to solvent from the cytoplasmic side (Figure 1B,C). Each protomer of Vc-NhaP2 has 13 transmembrane segments (TMSs) connected by short loops or helices on the membrane surface (Figure 1A). TMSs IV–VII and TMSs XI and XII form a six-helix bundle, while TMSs I–III and TMSs VII–X form the dimer interface. TMS V and TMS XII in the six-helix bundle are discontinuous [24], forming the distinctive NhaA fold initially identified in Ec-NhaA Na^+^/H^+^ antiporter [28,29]. In the *E. coli* antiporter, the NhaA fold is crucial for the conformational change expected to occur upon ligand binding [28]. The NhaA fold is shared by multiple members of the CPA1 and CPA2 family, e.g., the NhaA of *E. coli* (Ec-NhaA) [29], NapA of *T. thermophilus* (Tt-NapA) [30], NhaP of *Pyrococcus abyssii* (Pa-NhaP) [18], and *Methanococcus jannaschii* (Mj-NhaP1) [19]. Besides possessing the NhaA fold, CPA transporters are often classified based on their ion selectivity and electrogenicity [31]. Both Ec-NhaA and Tt-NapA have been suggested to be electrogenic, exchanging one alkali cation for two protons [28,30]. Two conserved aspartates in the ion binding site of Ec-NhaA, Asp163, and Asp164 (so-called “DD motif”) located on TMS V have been proposed to be the primary proton carriers [29,31]. According to an alternative antiport mechanism for electrogenic Ec-NhaA, Asp163 forms a salt bridge with Lys300, in its protonated form. Ion binding breaks this salt bridge, allowing the bound ion to be alternatively transported either to the cytoplasm or periplasm [32]. The conserved DD motif and the salt bridge between Asp163 and Lys300 are essential for electrogenic antiporter function as disruption of the salt bridge renders electrogenic Ec-NhaA electroneutral [31,32]. 

## 3. Amino Acid Residues Affecting the Ion Binding and Selectivity of Vc-NhaP2

**Figure 2:**
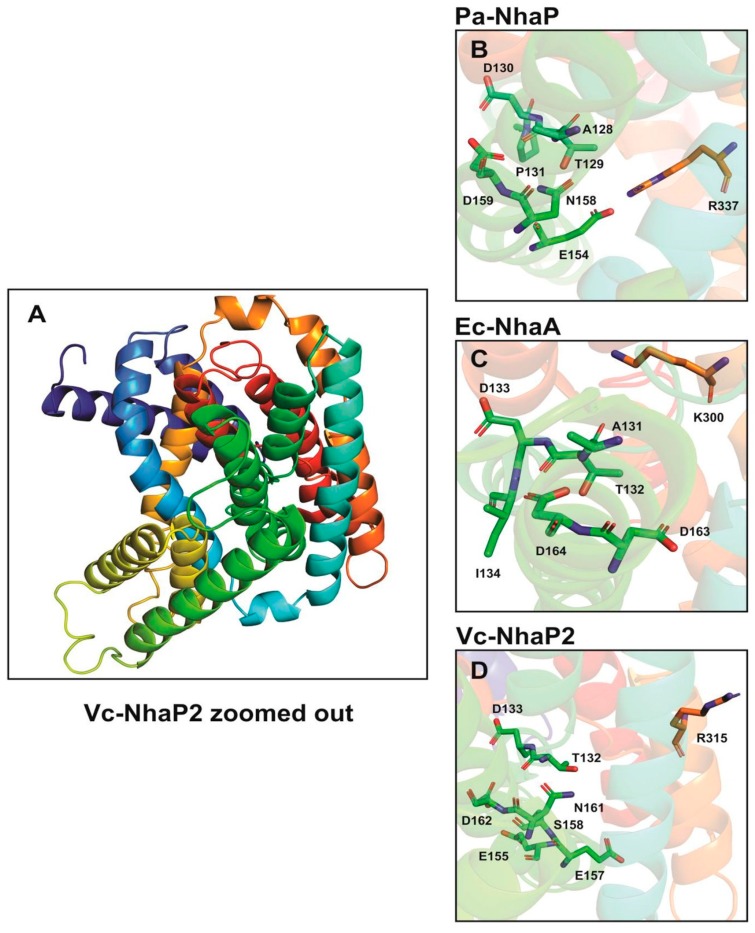
Amino acid residues implicated in ion binding in NhaP-type antiporters. Structural data for proteins from NhaP2 of *V. cholerae* (Vc-NhaP2) (based on data from [15]) (**A**,**D**), *Pyrococcus abyssii* (Pa-NhaP) (PDB accession code: 4CZA) (**B**) and NhaA of *E. coli* (Ec-NhaA) (PDB accession code: 1ZCD) (**C**) were used to generate 3D images with PyMOL 1.6 [34]. Amino acid residues are shown in stick-and-surface representation.

In electroneutral CPA transporters, a highly conserved Asn-Asp motif (ND motif) is present instead of the DD motif and the Lys300 is replaced with an arginine [22,31]. In agreement with this, Vc-NhaP has the conserved ND motif (Asn161-Asp162) in TMS VI and Arg315 in TMS V (Figure 2) [22,24]. As determinants of ion selectivity, CPA transporters share eight highly conserved amino acid residues located on different transmembrane helices for either K^+^ or Na^+^ selective transport [31]. These residues form a compact group in the folded tertiary structure of the antiporters [22,31]. In the case of Na^+^/H^+^ antiporters belonging to CPA—e.g., Ec-NhaA—residues A131, the TD motif (Thr132-Asp133), and I134 on TMS IV together with a tandem Asp162-Asp163 on TMS V and Lys300 on TMS IX are involved in forming a structural motif determining the Na^+^ selectivity (Figure 2). In contrast, in CPA2—e.g., Pa-NhaP A128—the TD motif (Thr129-Asp130) and Pro131 on TMS V together with the ND motif (Asn158-Asp159) on TMS VI and a salt bridge formed between Glu154 and Arg337 are involved in Na^+^/H^+^ exchange (Figure 2). 

Antiporters showing K^+^ selectivity are suggested to contain polar or charged residues instead of the nonpolar residues adjacent to the TD motif [31]. This is also supported by our findings obtained from the combined in silico and mutagenesis analysis of the Vc-NhaP2 antiporter. We carried out two rounds of Ala mutagenesis on selected amino acid residues based on our in silico model predictions. We found that a cluster of negatively charged and polar residues belonging to TMS V and VI of Vc-NhaP2 form the cation-binding pocket in the middle of the membrane [24]. Glu155, Asp133, Thr132, and Ser158 from TMS V together with Asp162 and Asn161 from TMS VI, and the possible salt bridge partner of Glu157 and Arg315 are essential for K^+^/H^+^ transport (Figure 2). Vc-NhaP2 also contains a built-in filter in the vicinity of these conserved amino acid residues determining Na^+^, Li^+^, or K^+^ selectivity [24]. This has also been observed in the case of the eukaryotic antiporter from yeast *Zygosaccharomyces rouxii* (Zr-SOD22) that exports Na^+^ and Li^+^, but not K^+^ [33]. It has been suggested that a hydrophobic filter near the transporter’s ion binding site confers cation selectivity [33]. A triple Zr-Sod2-22 mutant, Thr141Ser-Ala179Thr-Val375Ile, was generated that gained K^+^/H^+^ transport capacity [33].

In the case of Vc-NhaP2 nine residues (Tyr151, Leu257, Glu258, Asn259, Asp273, Thr276, Gln280, Leu289, and Leu342) from TMS IX, X, and XII form a trans-membrane pathway for translocated ions with a built-in filter determining cation selectivity [24]. Limited Ala-scanning mutagenesis supported these predictions. Thus, mutations Asp162Ala and the nearby Asn161Ala inactivated the antiporter completely [22] (as one would expect after removal of putative ion-coordination ligands), while Asp273Ala, Thr276Ala, Leu289Ala, and Leu342Ala substitutions indeed resulted in drastic restrictive changes in K^+^/Na^+^ specificity [24]. Furthermore, alanines in positions 251, 257–258, and 280, brought in a completely new type of activity: direct Li^+^/H^+^ exchange [24]. These experimental results further validate our in silico modeling of Vc-NhaP2. We have also identified that mutation of a single Gly159 to alanine, located in the vicinity of the putative cation binding pocket enables Vc-NhaP2 to exchange Li^+^ for H^+^ directly [22]. Interestingly, any negatively or positive charged residue at the position of G159 allows Li^+^ to be exchanged for H^+^ [22]. The Gly159Ala mutant variant was also able to protect the antiport deficient *E. coli* strain (highly sensitive to Li^+^) against high concentration of Li^+^ (up to 250 mM) when expressed in trans [22]. 

This finding supports the idea of ‘ligand shading’ in the active center of Vc-NhaP2, when different alkali cations use overlapping but not identical sets of ligands, thus differently affecting the possibility of protonation of the antiporter during the catalytic cycle [4]. In the future, construction of double and triple Ala substitution will be attempted in order to eliminate all the ‘excess’ coordination ligands and thus manipulate the ion selectivity to yield strictly selective Na^+^/H^+^, K^+^/H^+^, or Li^+^/H^+^ antiporters, independently of the residues of TMS IX-XII, which comprise a distant ‘selectivity filter’. 

The Na^+^/H^+^ antiport cycle in *E. coli* NhaA is suggested to follow the ‘alternating-access’ mechanism of secondary active transporters, with the ion binding pocket either open to the cytoplasm or the periplasm [28,29]. Upon ligand binding, a conformational change is induced which involves rearrangement of TMSs IV, V, and XI in the core domain, allowing ions to be transported either to the cytoplasm or the periplasm [36]. NapA of *T. thermophilus* (Tt-NapA) and NhaP of *M. jannaschii* (Mj-NhaP1) and *P. abyssii* (Pa-NhaP) the alternating access has been suggested to follow a two-domain elevator mechanism for the Na^+^/H^+^ transport, where the core domain rotates against the fixed dimerization domain with the release of Na^+^ or H^+^ either to the cytoplasm or the periplasm [19,30,37]. In the case of Mj-NhaP1, only binding of ligands (Na^+^) has been shown to induce the conformational change and allow the ion binding pocket to alternatively access either the cytoplasmic or the periplasmic sides [19]. Recently, it has been suggested that a hydrophobic filter on the extracellular side controls ion accessibility to the binding pocket in Pa-NhaP, with the opening and closing of the gate strictly controlled by domain movement [37]. Microsecond MD simulations and transition-state sampling along with mutagenesis experiments revealed that the hydrophobic gate maintains a delicate balance between open and closed structures and without ions (Na^+^ or H^+^) it remains closed, thus preventing transmembrane cation leakage [37]. 

**Figure A1:**
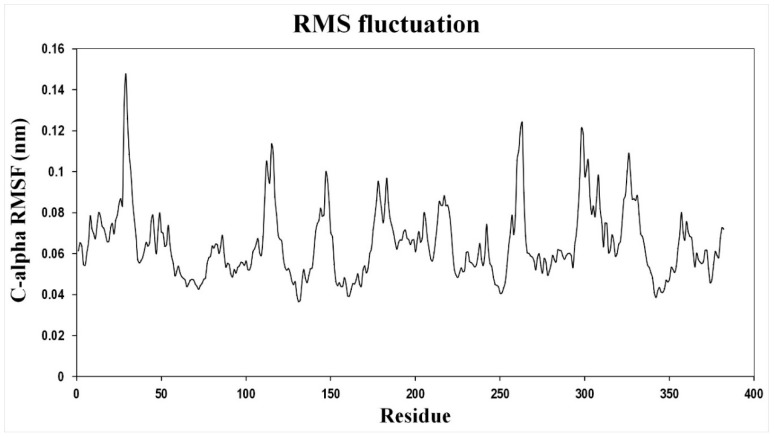
The root-mean-square fluctuations (RMSFs) of the backbone C-alpha coordinates are shown as a function of residue number over the 11 ns simulation. Residues 90–109, 129–142, 307–317, and 333–342 are located in the TMS IV, V, XI, and XII respectively. Residues 187–212 and 226–241 are located in the TMS VII and VIII respectively. All the other residues showing significant fluctuations are present in the loop regions. TMSs IV, V, XI, and XII form the core domain, whereas TMSs VII and VIII are a part of the dimerization domain.

In order to probe the conformational changes occurring upon ion binding in Vc-NhaP2, we carried out molecular dynamics (MD) simulations and the average fluctuation of the backbone C-alpha carbon for each residue was calculated over an 11 ns time scale. Amino acid residues in TMS IV, V, XI, and XII in the core domain and TMS VII and VIII in the dimerization domain showed the highest fluctuations (Figure A1). The amino acid residues forming the putative cation binding pocket did not show any significant fluctuations. This is in agreement with the findings that in Ec-NhaA, TMS III, IV, and XI in the core domain showed higher deuterium uptake upon Li^+^ binding in a hydrogen/deuterium exchange mass spectrometry experiment compared to TMS V containing the ion binding residues [36], suggesting that the conformational change occurring in the core domain alternatively opens the rigid ion binding pocket either to the cytoplasm or to the periplasm. These observations suggest that Vc-NhaP2 might follow the ‘alternating access mechanism’, where the major conformational change occurs in the core domain, especially in the cross-over region between the extended chains of TMS V and XII, and thus alternatively opens the ion binding pocket either to the cytoplasm or to the periplasm. For the MD simulations, an all-atom Vc-NhaP2 model generated using Robetta [24,27] was imbedded in the lipid bilayer and the water, lipid, and protein components were extensively energy minimized using Charmm-Gui protocols [38,39] and Gromacs 5.1.4 [40]. The total system consisted of about 120,000 atoms in an orthorhombic simulation cell with a free NaCl concentration of 250 mM. Equilibrium MD simulations were performed after energy minimization and 11 ns of equilibration with position restraints. All simulations were carried out under periodic boundary conditions at constant temperature (T = 310 K) and pressure (P = 1 bar). It is still not clear if Vc-NhaP2 follows a two-domain elevator access mechanism as suggested for Tt-NapA or Mj-NhaP1. Longer MD simulations and transition-state sampling will be carried out in the future to analyze the possible ion exchange mechanism and associated conformational changes for Vc-NhaP2.

## 4. Putative Role of the Soluble C-Terminus of NhaP2

The NhaP type antiporters from eukaryotes have long C-terminal tails that play an important role in the regulation of ion transport [41,42]. The human Na^+^/H^+^ exchanger, NHE1, consists of a N-terminal integral membrane domain involved in ion binding and a long C-terminal regulatory domain comprising ~300 cytoplasmic amino acids [41]. A number of signalling molecules are involved in the regulation of the C-terminal region which is linked to the N-terminal ion binding domain [43]. NHE1 is activated upon a calmodulin-dependent binding of Ca^2+^ to the NHE1 cytosolic C-terminal region. The NHE1 cytosolic C-terminal binding region has been crystallized in complex with calmodulin and Ca^2+^ [43]. Calmodulin binds to both a high-affinity region and to a low affinity region in the C-terminal domain. In the absence of calmodulin, the high-affinity binding site of NHE1 possibly interacts with the N-terminal transmembrane domain, acting in an auto-inhibitory manner [43]. NHE1 is activated in the presence of increased intracellular Ca^2+^ and calmodulin. Recently, it has been reported that the extracellular signal-regulated kinase (ERK) mediated phosphorylation of the C-terminal domain that is involved in the structural and functional changes of NHE1 [44].

In contract, the C-terminal cytoplasmic tails in prokaryotic antiporters are much shorter compared to the eukaryotic ones, e.g., the C-terminal tail of Vc-NhaP2 is predicted to consist of only 120 amino acids [45,46]. In prokaryotes, the C-terminal tail has been shown to play a role in the function and/or regulation of these antiporters. In cyanobacterial Syn-NhaP1, deletion of the C-terminal hydrophilic tail resulted in a dramatic decrease in Na^+^/H^+^ and Li^+^/H^+^ antiport activity [47]. The C-terminal deletion mutant of Vc-NhaP2 showed diminished K^+^/H^+^ and Na^+^/H^+^ antiport activity, with a 5-fold decrease in the affinity for its major substrate K^+^ [45,46]. When the truncated C-terminal deletion mutant of Vc-NhaP2 was expressed in an antiport deficient *E. coli* strain, it caused increased sensitivity of the *E. coli* host to Na^+^ ions at neutral pH [45,46]. Though the chromosomal C-terminal deletion mutant of Vc-NhaP2 did not affect the ability of *V. cholerae* to grow at high potassium concentrations at acidic pH 6.0, the kinetic analysis clearly indicated that the cytoplasmic portion of Vc-NhaP2 is required for its optimal ion binding and maximal activity [45,46].

Interestingly, the C-terminal tail of Vc-NhaP2 is predicted to contain significant domain structure, including the Rossman fold that binds to dinucleotide cofactors such as NAD and FAD [46]. The Rossman fold has been found in K^+^ channels and is capable of pH induced conformational changes in protein upon cofactor binding [48]. The domain is suggested to be involved in pH-sensitive gating of K^+^ channels [48]. Dynamic light scattering experiments at pH 7.0 with FAD indicated that FAD stabilized the C-terminal region of Vc-NhaP2 in an oligomeric form compared to experiments conducted in the absence of FAD [46]. More analysis is needed to fully understand the possible involvement of the C-terminal domain in the regulation of ion transport of Vc-NhaP2. The X-ray structure of the Vc-NhaP2 C-terminal tail, cross-linking, and mutagenesis experiments will be the first step towards understanding the possible regulatory role and also the in situ role of the C-terminal tail in facilitating the oligomerization of the antiporter. 

## 5. NhaP Paralogues in Acid Tolerance Response (ATR) of Vibrio Cholerae

*V. cholera* is transmitted by the fecal–oral route. It must pass through the low pH environment of the stomach to reach the small intestine where it colonizes in the intestine and secretes cholerae toxin causing cholera diarrhoeal disease [49]. It possesses an acid tolerance response (ATR) that would increase its survival within these hostile environments [50,51,52,53]. ATR is suggested to be a “significant factor in their epidemic proliferation and virulence” [50,51,52,53]. This survival system has been shown to be composed of a complex cascade of proteins, among which are several inducible amino acid decarboxylases. An essential component of organic and inorganic ATR in *V. cholerae* is the *cadABC* system. *cadA* encodes an inducible lysine decarboxylase [50,51,52,53]. *cadA* was shown to be the second gene in an operon with *cadB*, encoding a lysine/cadaverine antiporter. *cadC*, which belongs to the ToxR-like family of transcriptional regulators positively regulates transcription of *cadBA*. *CadC* is activated at low pH by AphB, a LysR-type activator by cooperating with the quorum-sensing-regulated activator AphA at the *tcpH* promoter on the *Vibrio* pathogenicity island (VPI) [53].

Our findings suggest how the Vc-NhaP group might boost the chances of survival when ingested *V. cholerae* cells pass the gastric acid barrier in the course of a normal infectious process [5], where the cells face the stressful conditions of low pH and high potassium concentrations [54,55]. The low pH is due to the secretion of H^+^ by the parietal cells into the stomach lumen up to 150 mmol/L [54,55]. H^+^ is pumped into the lumen via the K^+^/H^+^ ATPase that pumps out 1 H^+^ in exchange for one K^+^ ion and the K^+^ is then recycled by K^+^ channels [54,55]. Bacteria adopt different survival strategies to overcome the acidity. This is supported by the observation that wild type *V. cholerae* survived better compared to the Vc-NhaP123 triple deletion mutant when exposed to inorganic acid challenge [5]. In these experiments, *V. cholerae* cultures were resuspended in potassium-rich LBK medium adjusted to pH 3.5, 4.0, or 4.5 and incubated for different time intervals. Aliquots of cells were taken at each time point and spread onto LBK agar (pH 7.5) for a standard colony count. Furthermore, introduction of *nhaP*1, 2, and especially 3 genes in trans boosted the survival of mutant *V. cholerae* cells under these experimental conditions of inorganic acid challenge (unpublished observations).

## 6. Regulation of Vc-NhaP Isoforms

In vivo, the Vc-NhaP type antiporters function as a whole to maintain potassium homeostasis in the bacterial cytoplasm [5]. All three paralogues are crucial for survival of *V. cholerae* at acidic (pH 6.0) and alkaline (pH 8.0) pHs. Interestingly, the Vc-NhaP1 alone is efficient for the restoration of growth of *V. cholerae* at high and low potassium concentration at acidic pH 6.0, possibly due to its role in alkalinization of the cytoplasm of *V. cholerae* growing in acidic media [5,23] as it has been shown that the internal pH homeostasis mediated by Vc-NhaP1 is K^+^-dependent [23].

It is interesting to note how the three different Vc-NhaP paralogues situated at different locations on the chromosome are able to function in concert to maintain potassium homeostasis in the bacterial cytoplasm. It is possible that they are regulated in a quorum sensing-dependent manner like the *cadABC* and the *ivr* gene in *V. cholerae* [53]. Both the *cadABC* and *ivr* genes are activated in response to low pH and regulated by a signal transduction event in response to lower cell density. The regulation of the *ivr* gene encoding the Cl^−^/H^+^ antiporter is finely tuned in response to pH by AphB, which gets activated by AphA in a quorum sensing-dependent manner. At low pH, the *ivr* gene is activated and participates in the ATR response of *V. cholerae*. In the small intestine when *V. cholerae* is exposed to alkaline pH, the *ivr* gene is turned off to prevent excessive alkalinisation of the cytoplasm [53]. The regulatory proteins ToxR, ToxT, and TcpP that are involved in the virulence genes expression in *V. cholerae* are also regulated in a quorum sensing-dependent manner via AphB and AphA [56]. It is possible that Vc-NhaP paralogues are also regulated in a pH and quorum sensing dependent manner. The role of *AphB* and *AphA* in the regulation of Vc-NhaP paralogues is a subject for future investigation.

## 7. Perspective and Expected Impact of the Vc-NhaP Paralogues Research

Cation–proton antiporters are ubiquitous membrane transporters and the most universal transporting component in all living organisms studied so far. This is particularly evident when looking at the NhaP type antiporters due to their vast diversity in both eukaryotes and prokaryotes. This group of antiporters has evolved to play a role in a variety of physiological functions of all organisms due to their ability to mediate rapid cation/H^+^ exchange that makes them very efficient in enhancing the survival potential of the microorganism at different stressful environmental conditions, as well as in the human body. Studying the NhaP type antiporters in the dangerous human pathogen *V. cholerae* not only helps us to understand the physiology, structure, and dynamics of other prokaryotic homologues, it has also shed light on the possible structures and functions of its eukaryotic homologues. 

Antiporters of the Vc-NhaP type emerge as molecules that might be important in the early stages of infections caused by *Vibrios*. Therefore, all of these antiporters, but especially Vc-NhaP2, are prospective targets for the development of novel antimicrobials targeting this specific group of pathogens. The potential of cation–proton antiporters in general and NhaP type antiporters in particular as targets for antimicrobials remains virtually uninvestigated. We however feel that this potential is quite significant. Noticeably, in the dangerous human pathogen *Yersinia pestis*, elimination of genes encoding NhaA and NhaB sodium-proton antiporters resulted in complete loss of virulence in an in vivo model of plague; introduction of the *nhaA* or *nhaB* genes in trans restored the virulence of the *Y. pestis* mutant [57]. The *Y. pestis* strain with the deleted *nhaA* and *nhaB* genes survived very poorly in blood and blood serum ex vivo, as well as in artificial growth media containing Na^+^ levels and pH values similar to those of blood [57]. Thus, the Na^+^/H^+^ antiport is crucial for the survival of *Y. pestis* in the bloodstream of infected organisms and thus appears to be a promising drug target not only for *Y. pestis* but possibly other blood-borne bacterial pathogens. Given the possible role of Vc-NhaP2 in the ATR of *V. cholerae* outlined above, we hypothesize that the inhibition of Vc-NhaP2 alone (or, perhaps, of all three Vc-NhaP paralogues) might disrupt the infectious process caused by this pathogen as it attempts to cross the gastric acid barrier. One could also mention that the inhibition of antiporters of NhaP type (which are not widely represented in the genomes of benign gut microflora) is not as indiscriminative as the application of conventional antibiotics and thus should be less damaging to human microbiota in a real clinical setting. Future studies of Vc-NhaP-deficient strains in the in vivo models of cholera will further clarify a potential value of NhaP type antiporters as drug targets. One may expect that such novel remedies will be especially valuable against strains resistant to currently used antibiotics. This would have immediate medical applications (*V. cholerae*, *V. parahaemolyticus*, etc.) as well as industrial ones, e.g., in fish farming, where infections caused by a number of *Vibrio* species are among the most long-standing problems [58]. Results of studies focused on the 3D-structure and functioning of the NhaP-type antiporters will be important for the future development of inhibitors targeting these ion exchangers. 

The Vc-NhaP trio of antiporters represents a compact phylogenetic entity undergoing rapid divergent evolution. Using model-assisted scanning mutagenesis, we intend to investigate if the accumulation of seemingly neutral mutations, such as G159A, might be one of mechanisms of such divergent evolution. Having identified novel mutations in Vc-NhaP2 that manipulate the selectivity of ion translocation [22,24], we hope to be able to shed new light on the events in the active site of NhaP-type antiporters. In particular, this is expected to guide an extensive experimental testing of the available in silico tools for the structural analysis. On a more general note, one could expect that the results of experimental probing of the applicability of the *Phyre^2^* [26] and Robetta [27] models to cation–proton antiporters would be especially valuable for researchers studying this class of hard-to-crystallize proteins. From a methodological point of view, the reviewed studies would permit evaluation of the potential of comparative in silico modelling for structural analysis of ion transporters whose actual structures are not readily available (e.g., proteins that are important for practical applications but are hard to express and/or crystallize). 

## Abbreviations

ATR	Acid Tolerance Response
CPA	Cation Proton Antiporter family
FAD	Flavin Adenine Dinucleotide
MD	Molecular Dynamics simulation
NAD	Nicotinamide Adenine Dinucleotide
TMS	Transmembrane Segment
